# Evaluation of the Effect of Botulinum Toxin A on the Lymphatic Endothelial Cells

**DOI:** 10.1007/s00266-024-04061-7

**Published:** 2024-06-05

**Authors:** Mauro Vasella, Stefan Wolf, Lisanne Grünherz, Bong-Sung Kim, Nicole Lindenblatt, Pietro Giovanoli, Epameinondas Gousopoulos

**Affiliations:** Department of Plastic Surgery and Hand Surgery, University Hospital Zurich, Raemistrasse 100, 8091 Zurich, Switzerland

**Keywords:** Botulinum toxin A, Botox, Lymphatic endothelial cells, Lymphatic capillaries, Lymphatic system, In vitro systems, Edema

## Abstract

**Introduction:**

Botulinum toxin A (BoTA) is a neurotoxin formed by Clostridium botulinum, with a broad medical application spectrum. While the primary effect of BoTA is on the muscles, the effects of BoTA in other systems including the blood vasculature have already been examined, revealing unexpected actions. However, no studies exist to the best of our knowledge regarding the potential effects of BoTA on the lymphatic vascular system, possessing a critical role in health and disease. Isolated human lymphatic endothelial cells (LECs) were cultured in dedicated in vitro culture systems. The analysis including imaging and cell culture approaches as well as molecular biology techniques is performed to examine the LEC alterations occurring upon exposure to different concentrations of BoTA.

**Materials and Methods:**

Human LECs were cultured and expanded on collagen-coated petri dishes using endothelial basal medium and the commercial product Botox from Allergan as used for all our experiments. Harvested cells were used in various in vitro functional tests to assess the morphologic and functional properties of the BoTA-treated LECs. Gene expression analysis was performed to assess the most important lymphatic system-related genes and pathways.

**Results:**

Concentrations of 1, 5 or 10 U of BoTA did not demonstrate a significant effect regarding the proliferation and migration capacity of the LECs versus untreated controls. Interestingly, even the smallest BoTA dose was found to significantly decrease the cord-like-structure formation capacity of the seeded LECs. Gene expression analysis was used to underpin possible molecular alterations, suggesting no significant effect of BoTA in the modification of gene expression versus the starvation medium control.

**Conclusion:**

LECs appear largely unaffected to BoTA treatment, with an isolated effect on the cord-like-structure formation capacity. Further work needs to assess the effect of BoTA on the smooth-muscle-cell-covered collecting lymphatic vessels and the possible aesthetic implications of such an effect, due to edema formation.

**Level of Evidence V:**

This journal requires that authors assign a level of evidence to each article. For a full description of these Evidence-Based Medicine ratings, please refer to the Table of Contents or the online Instructions to Authors www.springer.com/00266.

## Introduction

The neurotoxin botulinum toxin A (BoTA), formed by Clostridium botulinum, inhibits the secretion of neurotransmitters such as acetylcholine at neuromuscular junction inducing muscular paralysis [1]. This is achieved through the cleavage of the protein SNAP-25 which prevents exocytosis of synaptic vesicles [2]. BoTA is broadly used in the medical field for the treatment of muscle spasms, pain or primary hyperhidrosis and since more than 20 years in aesthetic procedures to reduce the appearance of dynamic wrinkles [3–6]. Further uses include the treatment of overactive bladders [7], lower urinary tract disorders [8], Raynaud’s phenomenon [9], sialorrhea, [10] migraines [11], and trigeminal neuralgia [12]. It has even been successfully found to be safe in treating different pediatric conditions, like cerebral palsy [13, 14] or torticollis [15] or used as an adjunct in cleft lip treatment [16].

While the primary effect of BoTA is on the muscle level, it has been shown that BoTA may affect other tissues and their architectural elements as well, such as the fibroblasts, the adipocytes and the blood endothelial cells. Al-Qattan et al. have shown that BoTA might positively influence wound healing by limiting hypertrophic scarring [17]. This actions is thought to be mediated through hindering the proliferation and promoting apoptosis of the fibroblasts at the aging site, thus reducing fibrosis [18], without affecting the proliferation of normal human fibroblast distantly to the application site [19, 20]. Interestingly, procollagen type-I carboxy-terminal peptide levels were found significantly increased in fibroblasts grown in the presence of BoTA. Similarly, collagen type-I expression was found upregulated, along with a reduction of pro- and active-matrix metalloproteinase (MMP) 9 expression, leading to reduced collagen degradation rates [20]. The inhibition of fibroblast growth and proliferation was also observed by Xiao et al. in vitro using fibroblasts cultured from tissue samples. Furthermore, they noticed a decreased transforming growth factor-b1 (TGF-b1) which directly affects the connective tissue growth factor (CTGF) and in turn can cause excessive collagen deposition [21]. They concluded that BoTA not only inhibited fibroblast proliferation but also decreased CTGF expression within these cells, possibly further explaining the positive effect of BoTA in hypertrophic scarring [22]. Jeong et al. further underlined the inhibition of fibroblast proliferation in scar tissue samples, moreover, decreased a-smooth muscle actin protein and mRNA levels, and inhibited fibroblast-to-myofibroblast differentiation when applying BoTA to dermal fibroblasts from hypertrophic scar tissue samples in series of in vitro experiments [23]. These findings suggest that BoTA has the potential to affect dermal remodeling.

The adjuvant usage of low concentrations of BoTA in fat grafting was found to support adipocyte survival, an effect mediated through increased angiogenesis [19, 24, 25]. More specifically, BoTA was shown to upregulate vascular endothelial growth factor (VEGF) and CD31 (platelet endothelial cell adhesion molecule/PECAM-1) expression levels, thus resulting into increased sprouting of blood vascular endothelial cells (BECs) [24, 26]. A consistent positive effect of BoTA on the blood vasculature has been demonstrated through the reported vasodilation of venules and arterioles [27] or its protective role against the apoptosis of human dermal microvascular endothelial cells when exposed to ischemia and reperfusion [28].

In contrast, high concentrations of BoTA were shown by Gugerell et al. to exert the opposite effect on the blood vasculature, decreasing the formation of vessels by downregulating the expression of *VEGF-A (Vascular Endothelial Growth Factor A)* and *ANGPT2 (Angiopoietin 2)* [26]. High BoTA concentrations were also noted to negatively impact keratinocytes, leading to decreased proliferation capacity and migration activity. These results indicate that the effect of BoTA is not limited solely to the application point but can impact tissue components neighboring its application situs.

The extremely extensive use of BoTA worldwide, attributed to its widespread inclusion in aesthetic applications, coupled with its documented side-effects underlines our need to better map and understand the possible outcomes of BoTA application in different tissues. While circulation system possesses a rather prominent role and was logically among the first systems to be examined, the analysis was limited to the blood vasculature, neglecting completely the assessment of the lymphatic vasculature in response to BoTA. Understandably, the transparent nature of the lymphatic system and the delayed development of specific markers to study it contributed to this oversight. But the multiple and crucial roles of the lymphatic vasculature, ranging from tissue fluid homeostasis to immune surveillance and even aging [29], urge us to examine and understand the repercussions of BoTA usage in the function and capacity of the lymphatic vascular system as well. It is evident that the usage of BoTA is compromising the lymph drainage efficiency in the treated areas, as the reduced muscle contraction cannot readily propel and drain the lymph. Here, our motivation and aim were to examine if BoTA may directly modify the properties and function of the structural components of the lymphatic system itself, the LECs.

In the current study, we utilized variable in vitro culture systems and human LECs along with different concentrations of a broadly commercially BoTA. Functional tests, imaging analysis and gene expression analysis were used to examine potential morphological, functional and genomic alterations upon treatment with BoTA. Our results indicate that BoTA has a marginal and limited direct effect on the LECs and subsequently the lymphatic capillaries and any possible influence of BoTA on the lymphatic system should be indirect.

## Materials and Methods

### Cell Culture of Primary Lymphatic Endothelial Cells

Primary human lymphatic endothelial cells (LECs) were provided through the kind donation of Prof. Michael Detmar (Institute of Pharmaceutical Sciences, ETH Zurich). The LECs were isolated from neonatal human foreskin (kind donation from the laboratory of Prof. Michael Detmar, ETH Zurich, Switzerland) and cultured following the protocol described by a study conducted by Schulz et al. [30].

In brief, LECs were seeded and expanded on type I collagen-coated tissue dishes (50 μg/mL in PBS, PureCol, Advanced BioMatrix, USA) in endothelial basal medium (EBM, Lonza, Switzerland) supplemented with 20% fetal bovine serum (FBS, Gibco, Life Technologies, USA), 1% antibiotic-antimycotic solution (Gibco, Life Technologies, USA), 4 mM L-Glutamine (Gibco, Life Technologies, USA), 25 μg/mL N^6^,2′-O-Dibutyryladenosine 3′,5′-cyclic monophosphate sodium salt (cell-permeable cAMP analog, Sigma-Aldrich, USA) and 10 μg/mL hydrocortisone (Sigma-Aldrich, USA). The cells were maintained at 37 °C and 5% CO_2_ up to passage 13.

Experiments were performed in starvation medium (EBM with 2% FBS and 1% antibiotic-antimycotic solution) as a control, supplemented human recombinant VEGF-165 Protein (40 ng/mL, Thermo Fisher Scientific, USA) as a positive control or BoTA (botulinum toxin A, Allergan, Ireland).

### Proliferation Assay

The proliferation assay was performed by seeding 2 × 10^3^ LECs per well onto a 96-well glass bottom microtiter plate (Corning, USA) coated with type I collagen for 24 h in full supplemented EB Medium. Following 6 h of incubation in starvation medium (SM) the cells were incubated in reduced medium containing 1 Unit (U), 5 U or 10 U of BoTA or VEGF-A. After 24, respectively, 48, hours cells were incubated with 4-Methylumbelliferyl heptanoate (100 µg/mL, Sigma-Aldrich, USA) for 1 h and the fluorescence intensity, proportional to the number of viable cells, was measured at 355 nm excitation and 460 nm emission (SpectraMAX GEMINI EM, Molecular Devices).

### Monolayer Wound-Healing Assay

To conduct the scratch assay, LECs were seeded at a density of 8 × 10^4^ per well onto a 24-well tissue plate coated with type I collagen overnight in full supplemented EB Medium. After 6 h of starvation in starvation medium (EBM with 2% FBS and 1% anti-anti), one cross per well was scratched into the cell monolayer, using a bended pipette tip and in a standardized manner. Upon washing, the cells were incubated in starvation medium containing 1 U or 5 U of BoTA and VEGF was used as positive control. Pictures were taken from the center of the cross for each well at 5x and10x magnification for the timepoints 0 (right after addition of treatment) and 24 (24 h after treatment) with a Zeiss Axio Vert.A1 microscope. The open wound area was quantified with ImageJ software 1.52A.

### Cord Formation Assay

To perform the cord or “tube” formation assay, LECs were seeded at a density of 8 × 10^4^ per well onto a 24-well tissue plate coated with type I collagen and incubated overnight in full supplemented EB Medium. Following a 6-h starvation step in starvation medium (EBM with 2% FBS and 1% anti-anti), the cell monolayer was overlaid with a neutralized, isotonic collagen solution (2,5 mg/mL type I collagen in PBS (Gibco, Life Technologies, USA) adjusted to pH 7.4 with NaOH (Sigma-Aldrich, USA) in starvation medium containing 1 U or 5 U of BoTA and VEGF-A. After incubation for 24 h at 37 °C, representative pictures per well were captured at 10x magnification (Zeiss Axio Vert.A1 microscope) and the total length of the tube-like formation was measured with ImageJ software 1.52A.

### RNA Extraction and Reverse Transcription-Quantitative PCR (RT-qPCR)

LECs were seeded in a coated 6-well tissue dish in full supplemented EB Medium for 24 h. After starving for 6 h in reduced medium (EBM with 2% FBS and 1% anti-anti), cells were incubated for 24 h with reduced medium containing 1 U and 5 U of botulinum toxin A using VEGF recombinant human Protein as positive control (40 ng/mL).

RNA was extracted using NucleoSpin XS RNA isolation kit (Macherey Nagel, Germany) according to the manufacturer`s guidelines, and cDNA was synthesized through reverse transcriptase using cDNA high-capacity kit (Thermo Fisher Scientific, USA). Quantitative PCR was performed with QuantStudio 5 (Applied Biosystems, USA) using Fast SYBR Green Master Mix (Gibco, Life Technologies, USA). The expression levels of distinct lymphatic endothelial genes were determined by applying the ΔΔct method using the primers listed in Table 1.

**Table 1:** List of employed RNA primers for RT-qPCR

Primer	Forward 5’ → 3’	Reverse 5’ → 3’
B2M	TGTGCTCGCGCTACTCTCTCT	CGGATGGATGAAACCCAGACA
VEGF-A	CTACCTCCACCATGCCAAGT	GCAGTAGCTGCGCTGATAGA
VEGF-C	CACCACCAAACATGCAGCTG	TGAAAATCCTGGCTCACAAGC
VEGF-D	ATGGACCAGTGAAGCGATCAT	GTTCCTCCAAACTAGAAGCAGC
PROX1	ACAAAAATGGTGGCACGGA	CCTGATGTACTTCGGAGCCTG
LYVE-1	AGCTATGGCTGGGTTGGAGA	CCCCATTTTTCCCACACTTG
PDPN	AGGCGGCGTTGCCAT	GTCTTCGCTGGTTCCTGGAG

### Statistical Analysis

All experiments were conducted at least in triplicates. The statistical analysis was performed using GraphPad Prism V 8.0 (GraphPad Software, San Diego, CA, USA). All data represent the mean ± SD. Results from all treatment groups were compared to starvation medium group using a one-way ANOVA followed by a Dunnett’s post hoc test. *P* values < 0.05 were considered significant.

## Results

### BoTA did not Influence the Proliferation Capacity of Lymphatic Endothelial Cell In Vitro

**Fig. 1:**
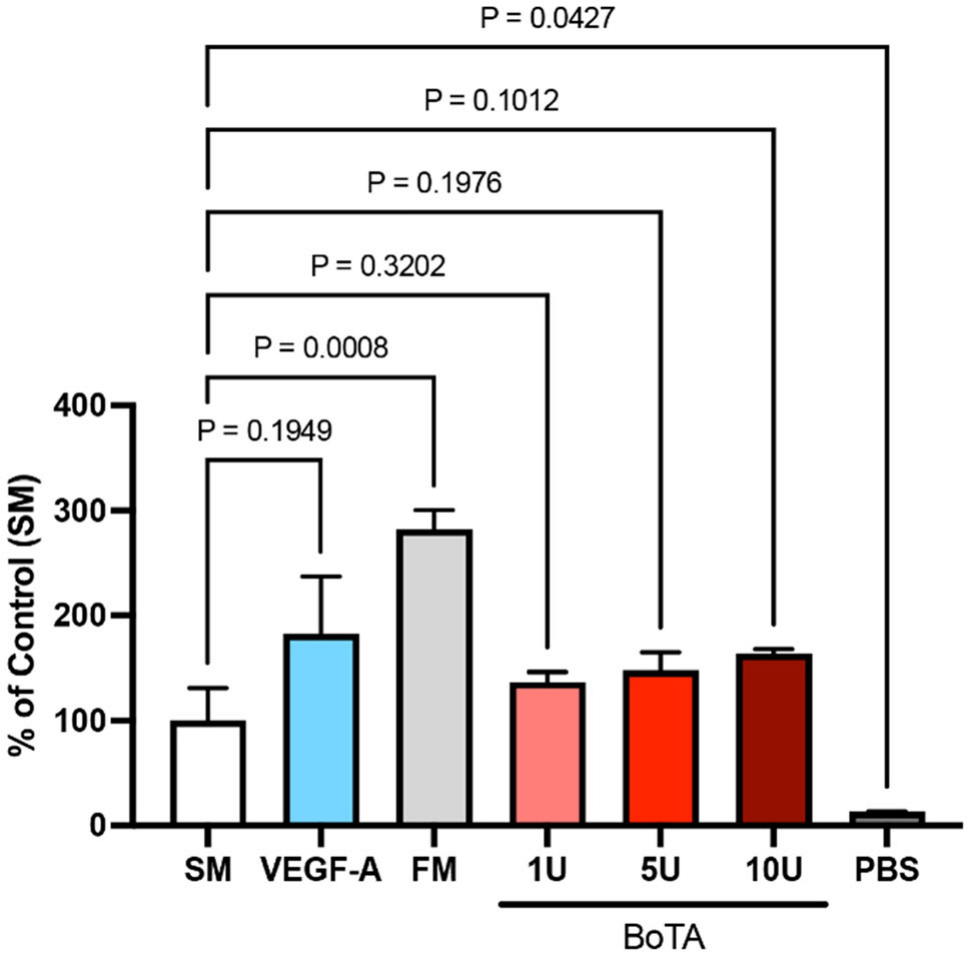
BoTA treatment did not alter LEC proliferation in vitro. **A** The proliferation capacity of LECs was assessed using 4-methylumbelliferyl heptanoate, while 1 U, 5 U, and 10 U of Botox did not alter the proliferation compared to the starvation medium (SM). As a positive control full medium (FM) induced LEC proliferation, while PBS alone reduced proliferation (experiment conducted in triplicate and one representative experiment is presented; *N* = 3–4, significance analyzed with one-way ANOVA and the Dunnett’s post-test)

To investigate the direct effect of BoTA on the LECs, we compared the proliferation of LECs upon exposure to difference BoTA concentrations, along with all additional appropriate controls (starvation medium, full medium, VEGF-A in a starvation medium (SM) and PBS alone). The dosing of 1 U, 5 U and 10 U was selected based on the most common range of unit injections in patients. Our analysis indicated that only the full culture medium (FM) resulted in increased LEC proliferation, while PBS (negative control) exhibited the least proliferation (SM: 100 ± 31.33%; VEGF-A: 182.5 ± 54.78%; FM: 282.1 ± 18.55%; 1 U: 137.0 ± 9.12%; 5 U: 148.0 ± 16.83%; 10 U: 163.4 ± 4.41%; PBS: 13.12 ± 0.35%). The proliferation rates across all BoTA doses did not reveal any significant alternations, suggesting that BoTA does not directly influence the proliferation or apoptosis of treated LECs. The results are depicted in Fig. 1.

### BoTA does not Alter Migration of LECs

**Fig. 2:**
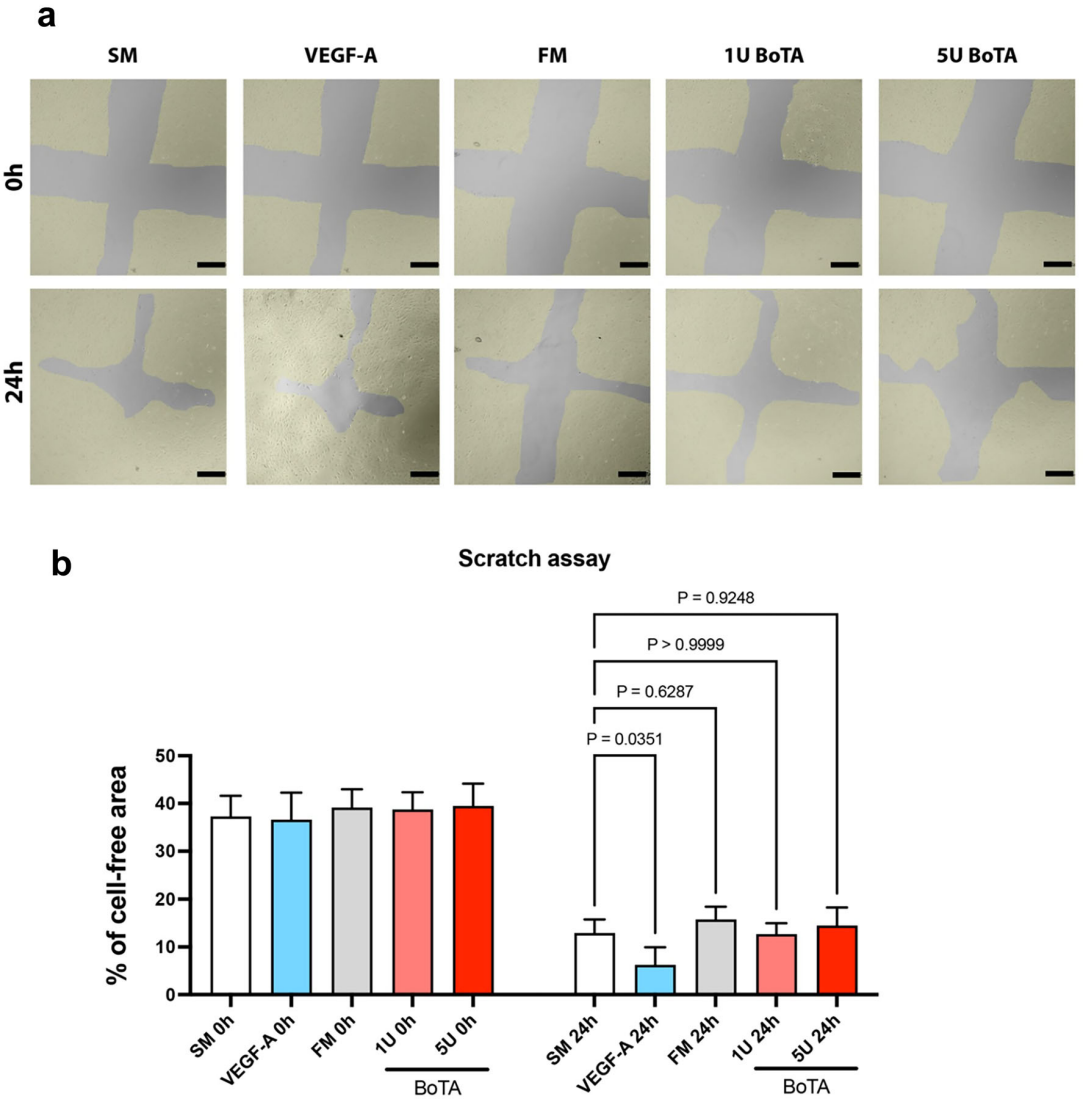
BoTA did not influence the migration potential of cultured LECs. **a** Representative picture of a wounded LEC monolayer at 0 h and after 24 h across all different conditions. The yellow area indicates the LEC monolayer. **b** VEGF-A significantly increased LEC migration compared to starvation medium (SM). Fully supplemented medium (FM) and BoTA treatment regardless of concentration used did not alter LEC migration. (Experiment conducted in triplicate; here we present one representative experiment; *N* = 4, significance analyzed with one-way ANOVA and the Dunnett’s post-test)

LEC migration was assessed using the well-established and reliable wound-healing assay. After 24 h of incubation, the VEGF-A group which functioned as a positive control indeed showed the smallest percentage of open area. The starvation medium (SM) had a significantly larger open area when compared to the VEGF-A group. The FM and BoTA medium groups (1 U and 5 U) did not show a significant difference compared to SM (values indicate % of cell free area: SM: 12.87 ± 2.87%; VEGF-A: 6.23 ± 3.73%; FM: 15.71 ± 2.69%; 1 U: 12.68 ± 2.30%; 5 U: 14.49 ± 3.80%), indicating that BoTA as an external chemical signal does not modify the LECs migration capacity. Images and results of the scratch assay are summarized in Fig. 2.

### BoTA Influences Lymphangiogenesis by Significantly Decreasing the Maximum Tube Length of Cultured LECs

**Fig. 3:**
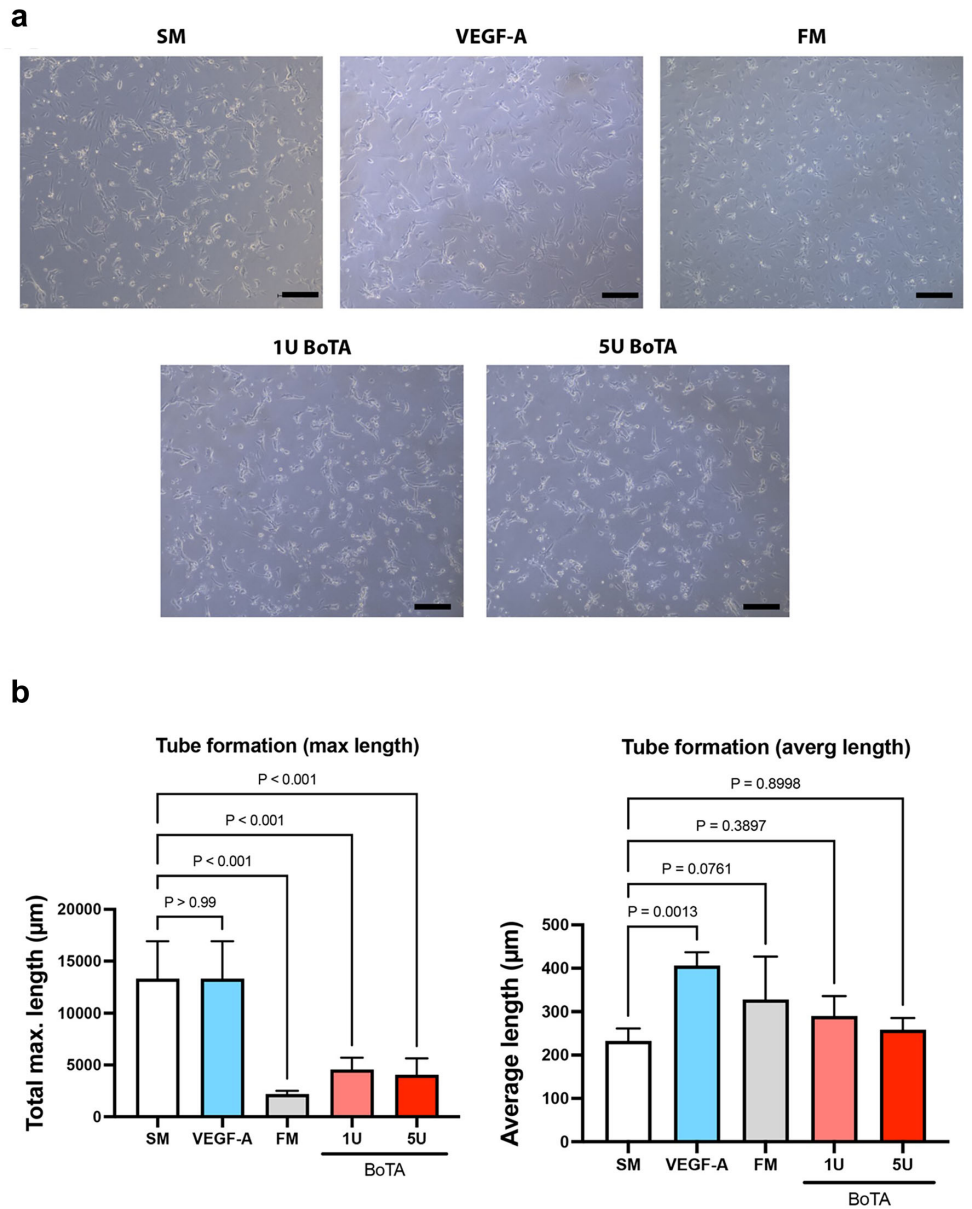
BoTA reduced the maximum length of LEC cord formation, while average length remained unaffected. **a** Microphotographs depicting the 24-h incubation timepoint of LECs, which has been overlaid with a type I collagen gel containing VEGF-A or BoTA. **b** BoTA and FM significantly reduced the maximum tube length compared to SM. The average tube length was significantly increased in the presence of the positive control VEGF-A., while BoTA conditions appear comparable to SM. (Experiment conducted in triplicate; here we present one representative experiment; *N* = 4, significance analyzed with one-way ANOVA and the Dunnett’s post-test)

The cord/tube formation assay is used to model the re-organization stage of lymphangiogenesis. The maximum length (measured in µm) of the tube formation was found to be the highest in the negative (starvation medium) and positive control (VEGF-A). The maximum length was significantly reduced in the FM and both BoTA conditions (max. length: SM: 13308 ± 3610 µm; VEGF-A: 13402 ± 2480 µm; FM: 2185 ± 318 µm; 1 U: 4557 ± 1157 µm; 5 U: 4064 ± 1584 µm). The average tube length was significantly increased in the VEGF-A medium (average length: SM: 232.7 ± 28.84 µm; VEGF-A: 206.0 ± 30.93 µm; FM: 327.7 ± 99.25 µm; 1 U: 290.4 ± 45.18 µm; 5 U: 258.4 ± 27.14 µm). A trend for increased average tube length was also noticed in the FM and BoTA mediums, without reaching statistical significance (Fig. 3a and b).

### BoTA Did not Influence the Expression of Lymphatic-Related Genes in Treated LECs

**Fig. 4:**
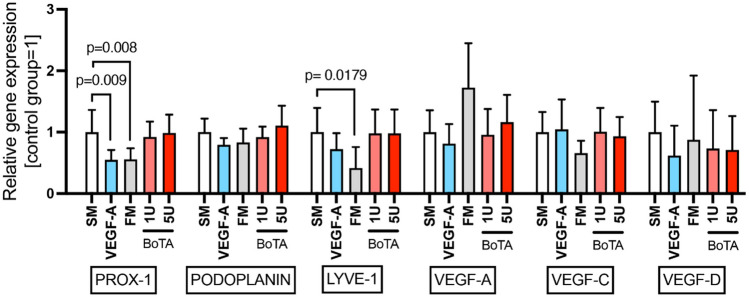
BoTA-treated LECs did not exhibit any lymphatic gene signaling alteration. PROX-1 expression was significantly reduced in response to VEGF-A and in FM, while LYVE-1 expression was reduced in FM. (Experiment conducted in triplicate; *N* = 6, significance analyzed with one-way ANOVA and the Dunnett’s post-test)

Gene expression analysis was used to determine how lymphatic signaling might be modified upon BoTA treatment. Interestingly, BoTA treatment in either concentration did not influence the expression profile of any of the examined genes. Only the expression levels of PROX-1 and LYVE-1 were found significantly downregulated in the VEGF-A/FM and FM media, respectively, compared to the SM treatment PROX-1 expression: VEGF-A = 0.549-fold, FM = 0.556-fold and LYVE-1 expression: FM = 0.415-fold), as illustrated in Fig. 4.

## Discussion

BoTA finds multiple additional applications in the field of plastic & reconstructive surgery and beyond, due to its capability to relax striated muscle tissue through inhibition of acetylcholine release. Thus, its use goes far beyond the reduction of dynamic mimic lines (wrinkles) and is effectively employed against a wide range of indications including an increase blood flow and support wound healing [31, 32]. Interestingly, BoTA has been anecdotally reported in the treatment and prevention of hypertrophy scars and keloids as well [18, 33–35] with the initially encouraging preliminary results lacking prospective clinical assessment and therefore the respective degree of evidence. One could hypothesize that the positive effect could be mediated through the BoTA-induced contraction of the fibroblasts, leading to reduction of cell length and eventually an improved mechanobiological milieu [36].

As the vast majority of the BoTA application affects the facial tissue that is particularly dense in blood and lymphatic vascularization particularly around the periorbital area, it has been striking that while a few reports examined the effects of BoTA on the blood vascular system, such studies have not been performed for the lymphatic vasculature.

The effects of BoTA on the blood vascular system are related to an increased blood vasodilation, as the toxin relaxes the vascular smooth muscle cells and inhibits the sympathetic input [37]. Similar effects have been reported in skin-flaps of preclinical models [27]. Park et al. were able to confirm these results and demonstrated that vessel which were pretreated with botulinum toxin B showed significantly increased diameter and blood flow velocity [38]. The vasoactive effect of BoTA was found to be mediated on the one hand through the increased expression of CD31, iNOS and CD31 which are significant players in proliferation of endothelial cells and vasodilation [39] and on the other hand through the regulation of calcium sensitization of smooth muscle tissue and the eNOS/sGC/cGMP pathway, that inhibits arterial vasoconstriction [40]. Importantly, Gugurell et al. indicated a clear dose-dependent effect of BoTA in terms of re-epithelialization and angiogenesis potential. He suggested that while low doses (1 to 10 IU/mL) had a pro-angiogenetic effect without influencing proliferation or migration of endothelial cells, the higher doses (20 IU/mL) led to a negative effect on the proliferation and migration of keratinocytes as well as on neo-angiogenesis, which correlated with significantly lower expressions of MMP9, VEGF-A and ANGPT2 in endothelial cells [26].

The aforementioned results trigger naturally the question, whether similar effects are exerted on the lymphatic vascular system as well; a question that has not been addressed experimentally thus far. It is well established that the muscle tone and activity present the main drivers of lymph transportation. Logically, as BoTA paralyses for instance the facial mimic muscles for aesthetic purposes, it inhibits the muscle tone as driver of the lymphatic pumping activity. This is in line with reports mentioning increased puffiness, particularly in older patients with already compromised muscle function after receiving BoTA treatment [41]. Therefore, a potential negative effect of BoTA directly on the structural components of the lymphatic system, the LECs, could have critical implications, particularly in aging and increasingly photodamaged skin.

To examine this intriguing question, whether BoTA may exert a directly effect on the LECs, we evaluated the effect of a clinically relevant range of BoTA concentrations in various in vitro systems (proliferation, scratch-test/wound-healing test, cord formation test), to examine the behavior, function and response of the LECs to BoTA exposure. Our results demonstrate that BoTA significantly reduced the maximum tube length of LECs which could suggest decreased lymphangiogenesis and could possibly be a relevant factor influencing the adverse effects mentioned above. Furthermore, the average tube length in the BoTA groups showed a trend to increase, without reaching statistical significance. Therefore, we hypothesize that these observed effects balance themselves out and that BoTA should not have adverse effects on the LECs. Nevertheless, further studies including relevant in vivo models and multiple injections over a longer time would have to be performed to follow up on this hypothesis. Additionally, an analysis of well-established lymphatic markers, including secreted cytokines, maintenance and survival genes, was performed in an effort to understand if lymphatic signaling may be affected upon BoTA. Our results suggest that BoTA does not influence lymphatic gene expression in the studied in vitro culture systems versus the starvation medium control. However, further investigations would have to be conducted in relevant in vivo preclinical models.

The current work has been limited by the employment of in vitro systems only to evaluate the effect of BoTA on the lymphatic endothelial cells. Thus, we excluded the examination of the smooth muscle cell-covered lymphatic collectors; such an experimentation would necessitate either ethically questionable animal experimentation, or human lymphatic vessel explant culture, for which appropriate samples present an absolute limitation. The limitations are counterbalanced by the usage of human-derived LECs, the most relevant in vitro systems that enable us examine the process of lymphangiogenesis in detail, both morphologically and on a pathway level. Further work should attempt to examine the BoTA effects on the collecting lymphatic vessels, using appropriate models, in order to gain valuable information about the effect of the BoTA in more complex and biologically relevant setups, which are relevant in the field of reconstructive plastic surgery. Additionally, the objectification of the lymphatic vascular compromise (edema) that the BoTA achieves through the paralysis of the muscles responsible for the dynamic wrinkles would present a relevant study to better define the boundaries of esthetic interventions.

Despite the presence of only marginal and limited direct BoTA effects on the LECs, the transient but noticeable indirect effect of lymphatic dysfunction upon aesthetic BoTA interventions is gaining significant attention. Thus, a new era with efforts to identify lymphatic vessels activators has been commenced, for both pharmaceutical and cosmetic purposes [42].

## Conclusion

LECs were found to remain unaffected by BoTA, without any obvious phenotypic and functional restrain or aberrant gene expression profile. Further work should still attempt to explore and quantify the effect of BoTA-induced muscle paralysis in lymphatic dysfunction, as well as the limits of aesthetic BoTA applications, with a focus on the assessment of the generated lymphatic function deficits.
